# Prevalence and associated factors of foot ulcer among diabetic patients in Ethiopia: a systematic review and meta-analysis

**DOI:** 10.1186/s12889-019-8133-y

**Published:** 2020-01-10

**Authors:** Tadesse Tolossa, Belayneh Mengist, Diriba Mulisa, Getahun Fetensa, Ebisa Turi, Amanuel Abajobir

**Affiliations:** 1 Department of Public Health, Institutes of Health Science, Wollega University, P.O. BOX: 395, Nekemte, Ethiopia; 2 Department of Public Health, College of Health Science, Debre Markos University, Debre Markos, Ethiopia; 3 Department of Nursing, School of Nursing, Institutes of Health Science, Wollega University, Nekemte, Ethiopia; 4 African Population and Health Research Centre, Maternal and Child Wellbeing Unit, Nairobi, Kenya

**Keywords:** Diabetic foot ulcer, Associated factors, Ethiopia

## Abstract

**Background:**

Diabetes and its complications including foot ulcer constitute a global public health challenge attributing to a significant cause of morbidity and mortality. Foot ulcer is one of the long-term complication of diabetes mellitus which lead to infection and amputation of lower extremities. In Ethiopia, findings from few studies were inconsistent and there is a need to systematically pool existing data to determine the magnitude of foot ulcer in diabetics and factors contributing to it.

**Methods:**

We identified articles through electronic databases such as Medline, Hinari, Pub Med, Cochrane library, the Web of Science and Google Scholar. Accordingly, we identified 95 published and one unpublished article. Finally, eleven studies which fullfilled eligibility criteria were included in final systematic review and meta-analysis. Data were extracted using a standardized data extraction checklist and the analyses were conducted using STATA version 14. The Cochrane Q test statistic and *I*^*2*^ tests were used to assess heterogeneity.

**Results:**

The overall magnitude of foot ulcer was 12.98% (95%CI: 7.81–18.15) in diabetic patients in Ethiopia. Sub-group analyses revealed highest prevalence in Addis Ababa (19.31% (95%CI: 2.7. 41.37)). Foot ulcer was significantly associated with rural residence (OR = 2.72, 95%, CI: 1.84–4.01)), presence of callus on the feet ((OR = 12.67, 95%, CI: 6.47–24.79)), a body mass index of ≥24.5 ((OR = 2.68, 95%, CI: 1.58–4.56)), poor self- care practice ((OR = 1.47, 95%CI: 1.25–1.73)), type I diabetes mellitus ((OR = 0.42, 95%, CI: 0.22–0.79)), staying with DM for < 10 years ((OR = 0.23, 95%, CI: 0.11–0.50)), and age < 45 years ((OR = 0.44, 95%, CI: 0.21–0.92)).

**Conclusion:**

The prevalence of diabetic foot ulcers in Ethiopia is relatively low, although its trend is increasing from time to time. Socio-demographic factors, body weight, and healthcare practice contribute to the development of diabetic foot ulcers. Appropriate interventions towards patient self-care practice, lifestyle modification and follow-up are wanted to prevent diabetic foot ulcers.

## Background

Diabetes and its complications are becoming common global public health challenges attributing to a predominant cause of illness and death [1, 2]. There will be over 642 million people with diabetes globally by 2040. In 2018, the International Diabetes Federation (IDF) reported that about 4 out of 5 people aged 20–79 live with diabetes in low and middle-income countries accounting for 5–22% of global prevalence [3]. According to WHO’s estimation, in 2016 about 1.6 million deaths were directly caused by diabetes [4] with a lifetime incidence of foot ulcers occurring in one-fourth of diabetic patients [5]. In Sub-Saharan Africa, the complications of diabetes are more likely due to delay identification and poor management of cases. Proportions of patients with diabetic complications include retinopathy (27–66%), neuropathy (10–83%) and microalbuminuria (7–63%) [6].

Diabetic foot ulcer (DFU) is becoming more than an indicator of complication status, having an independent impact on lower-extremity amputation and mortality risk [7]. It is the main cause of infection and people with diabetes are 25 times more likely to need amputation than those without this metabolic condition [8]. It is also one of the complications of diabetes that can result in economic, social and public health burden, especially in low-income communities because it usually affects economically productive age groups, 30–45 years [9].

Similarly, about 3 million adults live with diabetes [10] and the prevalence of both microvascular and macrovascular complications has been increasing among diabetic patients in Ethiopia. Although the prevalence of foot ulcers in diabetic patients varies from place to place in the country, it ranges from 1.5–31.5%, which show a great variation across different geographical settings and times [11, 12]. Research also report different causes of foot ulcers in diabetic patients including older age, rural residence, poor self-care practice, staying with the disease for long years, high body mass index (BMI), type-2 DM, smoking, and presence of neuropathy [11–19]. However, there is no representative data on diabetic foot ulcer in Ethiopia. Therefore, this systematic review and meta-analysis aimed at estimating the prevalence of diabetic foot ulcer and to identify factors associated with a diabetic foot ulcer.

## Methods

### Search strategy

This systemic review and meta-analysis were conducted to assess the pooled prevalence and associated factors of diabetic foot ulcers among diabetic patients in Ethiopia. We checked the presence of systematic reviews and meta-analysis on this topic to prevent duplication. Both published and unpublished studies conducted since 2000 were searched thoroughly using electronic databases. These included Medline, Hinari, PubMed, Cochrane Library, Web of Science and Google Scholar. To find unpublished papers, some research centers, including the Addis Ababa Digital Library were used.

Pre-defined search terms were utilized to enable a comprehensive search strategy that included all the relevant studies. All fields within records and Medical Subject Headings (MeSH terms) were used to expand the search in advanced Pub Med search. The search strategy was prepared and modified for the various databases using important Boolean operators with initial keywords *(“diabetes mellitus” OR “diabetic foot ulcer” OR “complication of diabetics” AND “associated factors” AND “Ethiopia”).* The meta-analysis was reported using the Preferred Reporting Items for Systematic Reviews and Meta-Analyses (PRISMA) guidelines [20]. All searched literature was downloaded to Endnote (version X7.2,) to maintain and manage citations, and facilitate the review process.

### Selection and eligibility criteria

Studies that assessed the prevalence and determined potentially associated factors of diabetic foot ulcers that were written in the English language were included. The design of these studies was all observational study conducted in Ethiopia. Studies where there were difficulties in extracting necessary information and those studies published before 2000 were excluded because findings might be distorted due to changes in trend.

### Outcome measurement

There were two main outcomes. The primary outcome of interest was the prevalence of diabetic foot ulcers, which was estimated as the total number of diabetic foot ulcer cases divided by the total number of diabetic patients multiplied by 100. The second outcome was identifying factors associated with a diabetic foot ulcer in diabetic patients, which were determined using the odds ratio (OR) and calculated based on binary outcomes from the included primary studies. The major factors included in this review were age (< 45 years versus 45 years), sex (male versus female), types of diabetes (type I versus type II), residence (urban versus rural), self-care practice (poor versus good), duration of diabetes (< 10 years versus), BMI (< 24.5Kg/m2 versus 24.5 Kg/m2) and callus on the feet (absent versus present).

### Quality assessment and data extraction

Reference management software (endnote version X7.2) used to combine search results from databases and to remove duplicate articles. The Joanna Briggs institute meta-analysis of statistics assessment and review instrument (JBI-MAStARI) was used for critical appraisal [21]. Data were extracted by two data extractors (TT and DM) using a standardized data extraction checklist on Microsoft excel. For the first outcome (prevalence), the data extraction checklist included author name, year of publication, region (the area where studies were conducted), study design, sample size and number of participants with the outcome. For the second outcome (associated factors), data were extracted in a format of two by two tables, and then the log OR for each factor was calculated based on the findings of the original studies. Discrepancies between two independent reviewers were resolved by involving a third reviewer (BM) after discussion for possible consensus. AA has overseen the overall process of data extraction and synthesis

### Statistical analysis and synthesis

STATA version 14 statistical software was used to analyze the extracted data. The logarithm and standard error of the OR for each included study were generated using the “generate” command in STATA. Cochran’s Q test (reported as the *p*-value) and inverse variance index (I2) were used to check heterogeneity in the included studies. I2 values of 0, 25, 50, and 75% were considered as no, low, moderate, and high degrees of heterogeneity, respectively [22]. The high degree of heterogeneity was observed for the first outcome and thus a random-effects model was used to estimate the pooled prevalence. No heterogeneity was observed for six factors; hence, a fixed-effects model was computed. For the remaining three factors (sex, callus, and neuropathy) with moderate to a high degree of heterogeneity, the random-effects model was used to estimate the Der Simonian and Laird’s pooled effect. In addition, a meta-regression was conducted to identify the source of heterogeneity and there were no statistically significant results found to declare the presence of heterogeneity. A funnel plot of asymmetry was used to check the presence of publication bias. Furthermore, Egger’s statistical test was used to check the statistical significance of publication bias [23]. Subgroup analyses by region were carried out. Prevalence with 95% confidence interval (CI) and OR of the association between diabetic foot ulcer and factors in the form of forest plot was presented.

## Result

### Study selection

**Fig. 1:**
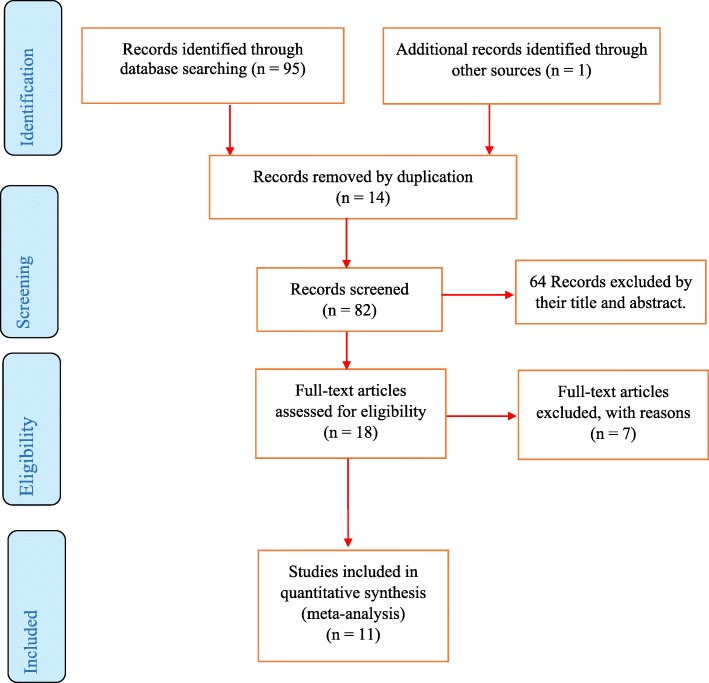
PRISMA flow diagram of included studies in the systematic review and meta-analysis of the prevalence of diabetic foot ulcer and associated factors among diabetic patients in Ethiopia, 2019

We identified 96 published and unpublished articles from different databases. Major reasons for excluded articles were duplication (14 articles) and mismatch with study objectives (64 articles). Eighteen articles were screened against eligibility criteria and only 11 studies scored 7 and above on the JBI quality appraisal criteria and included in the systematic review and meta-analysis (Fig. 1).

### Characteristics of included studies

Table 1 describes all cross-sectional studies included in the analyses. The total sample was 2768 diabetic patients, ranging from 108 to 418 [11, 12, 15–19, 24–27]. Studies from 4 regions and 1 town administrative of the country were included. Oromia [12, 16, 17], Amhara [19, 25, 26], and AA [11, 15, 27] contributing three studies in each region and 1 from southern nationalities [18] and 1 from Tigray [24] region (Table 1).

**Table 1:** Summary of Included Studies on prevalence of diabetic foot ulcers among diabetic patients in Ethiopia, 2019

S.n	Author	Year of publication	Region	Area	Study design	sample size	Prevalence (95%CI)
1	Esayas K Gudina et al. [16]	2011	Oromia	Jimma	Cross-sectional	108	10.19 (4.48, 15.89)
2	Tilahun AN, Waktola et al. [17]	2017	Oromia	Jimma	Cross-sectional	236	8.47 (4.92, 12.03
3	Alewiyu Yimam et al. [15]	2017	AA	AA	Cross-sectional	198	25.76 (19.67, 31.85)
4	Dawit Worku et al. [12]	2010	Oromia	Jimma	Cross-sectional	305	4.59 (2.24, 6.94)
5	M Gizaw, D Harries et al. [11]	2015	AA	AA	Cross-sectional	418	31.1 (26.6,35.54)
6	Kahsu Gebrekirestos et al. [24]	2013	Tigray	Mekele	Cross-sectional	228	12.28 (8.02,16.54)
7	Bedilu Deribe et al. [18]	2015	SNNP	Arbaminch	Cross-sectional	216	14.81 (10.08,19.55)
8	Asrat Agalu A et al. [25]	2013	Amhara	Dessie	Cross-sectional	216	1.85 (0.05, 3.65)
9	Kidist Reba L et al. [26]	2017	Amhara	Bahirdar	Cross-sectional	344	21.22 (16.9, 25.54)
10	Tesfamichael G et al. [19]	2017	Amhara	Gondar	Cross-sectional	279	13.62 (9.6, 17.64)
11	Hiwot Degu et al. [27]	2019	AA	AA	Cross-sectional	220	1.36 (0.17, 2.90)

Note: *AA*-Addis Ababa, *CI*-Confidence Interval, *SNNP*-Southern Nation Nationalities and people

### Prevalence of foot ulcer

**Fig. 2:**
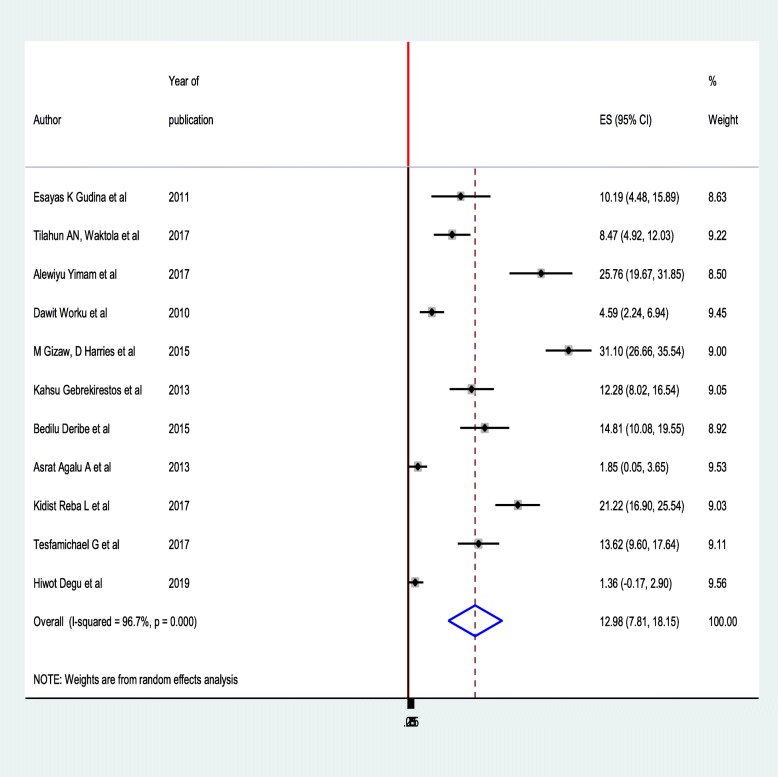
Forest plot of the pooled prevalence of diabetic foot ulcer among diabetic patients in Ethiopia, 2019

The pooled prevalence of diabetic foot ulcer was 12.98% (95%CI: 7.81**–**18.15). High heterogeneity was observed across the included studies (I^2^ = 96.7, *p* < 0.001). Both the highest (31.10% (95%CI: 26.66–35.54)) [11] and lowest (1.36%) [27] prevalence of diabetic foot ulcer was reported in Addis Ababa (Fig. 2). Sample size and year of publication were investigated to assess whether these were associated with heterogeneity using meta-regression models, although only region was statistically significant for underlying heterogeneity. Sample size (*p* = 0.374) and year of publication (*p* = 0.800) were insignificantly associated with heterogeneity.

### Subgroup analysis

**Fig. 3:**
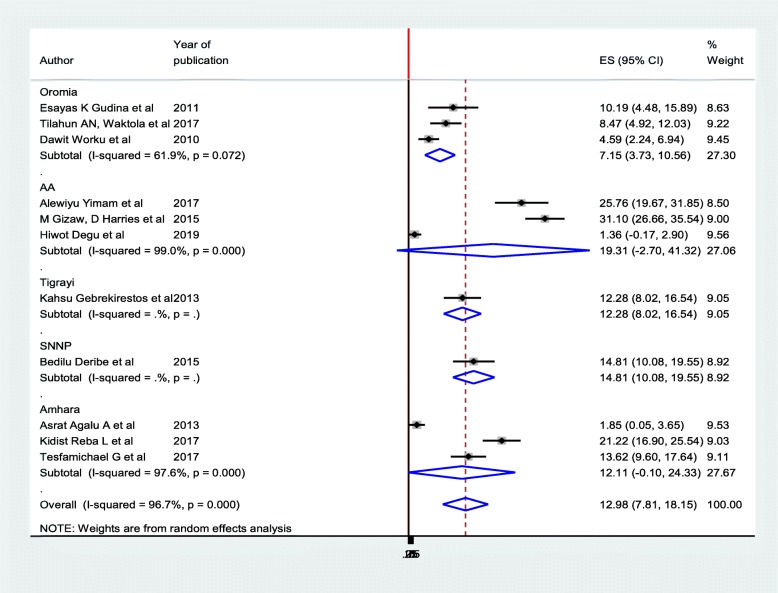
Subgroup analysis on prevalence of diabetic foot ulcer among diabetic patients in Ethiopia, 2019

The prevalence of diabetic foot ulcers was computed based on the regions where studies were conducted (Fig. 3). Accordingly, the prevalence ranged from 7.15% (95%CI: 3.73, 10.56) in Oromia region [12, 16, 17] to 19.31% (95%CI: 2.7. 41.37) in Addis Ababa [11, 15, 27]. The funnel plot was asymmetry, and the Egger’s test also showed statistically significant publication bias (*p* = 0.001) at 5% significant level (Table 2).

**Table 2:** Egger test and Begg’s test to see publication bias

Eger test	0.001
Begg’s test	0.013

### Sensitivity analysis

To identify a single study influence on the overall meta-analysis, sensitivity analysis was performed using a random-effects model and the result showed that there was no strong evidence for the effect of a single study on the overall meta-analysis result. The table showed that the estimates from a single study is closer to the combined estimate which implies the absence of a single study effect on an overall study (Table 3).

**Table 3:** Sensitivity analysis for single study influence on the overall study of diabetic foot ulcer prevalence in Ethiopia, 2019

S.n	Author	Sample size	Estimation	Lower limit	Upper limit
1	Esayas K Gudina et al. [16]	108	13.25	7.75	18.74
2	Tilahun AN, Waktola et al. [17]	236	13.45	7.80	19.11
3	Alewiyu Yimam et al. [15]	198	11.77	6.63	16.91
4	Dawit Worku et al. [12]	305	13.89	7.93	19.84
5	M Gizaw, D Harries et al. [11]	418	11.06	6.71	15.40
6	Kahsu Gebrekirestos et al. [24]	228	13.05	7.53	18.58
7	Bedilu Deribe et al. [18]	216	12.80	7.35	18.25
8	Asrat Agalu A et al. [25]	216	14.18	8.19	20.17
9	Kidist Reba L et al. [26]	344	12.13	6.98	17.29
10	Tesfamichael G et al. [19]	279	12.92	7.42	18.42
11	Hiwot Degu et al. [27]	220	14.23	8.39	20.07
Combined			12.98	7.80	18.15

### Factors associated with a diabetic foot ulcer in Ethiopia

#### Association between diabetic foot ulcer and residence

**Fig. 4:**
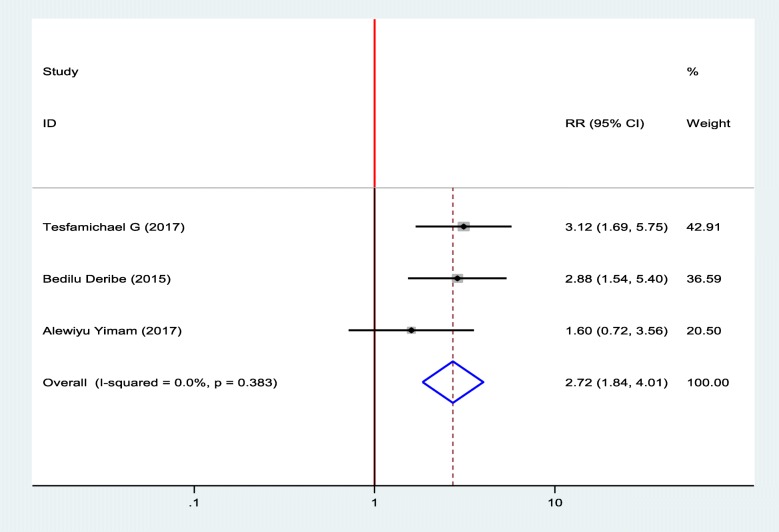
Forest plot of association between diabetic foot ulcer and Residence in Ethiopia, 2019

To identify the association between diabetic foot ulcer and residence, three studies were included in the meta-analysis [15, 18, 19]. Two of the included studies showed that being in rural was significantly associated with diabetic foot ulcers [18, 19] and one study showed that there was no association between residence and diabetic foot ulcers [15]. The pooled finding of the meta-analysis showed that living in rural was significantly associated with diabetic foot ulcer. Diabetic patients who were living in rural residence was 2.72 times more likely to develop foot ulcer as compared to diabetic patients who were live in an urban area (OR = 2.72, 95%, CI: 1.84–4.01). A fixed effect model was used hence, the included studies were not exhibited heterogeneity (I^2^ = 0.00%, *p* = 0.383) (Fig. 4).

#### Association between diabetic foot ulcer and callus on the feet

**Fig. 5:**
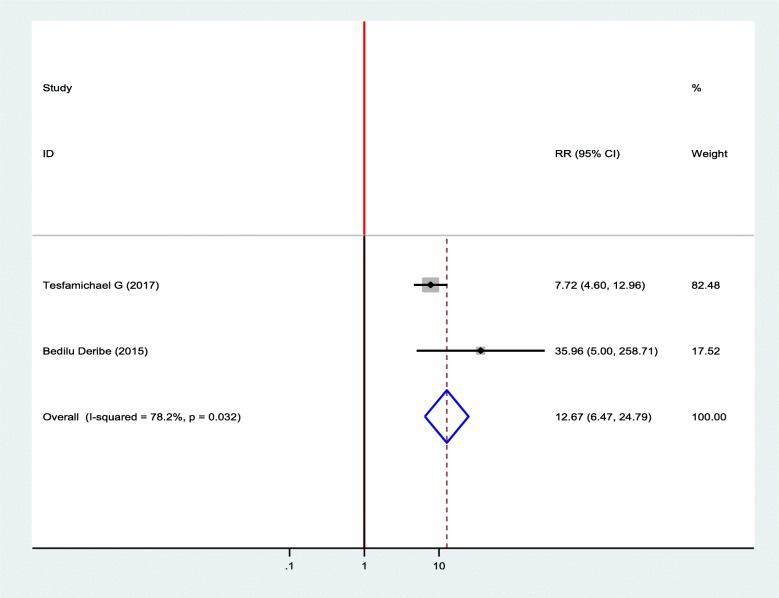
Forest plot of association between diabetic foot ulcer and callus on the feet in Ethiopia, 2019

Two studies were included in the meta-analysis to show an association between diabetic foot ulcer and callus on the feet [18, 19]. Accordingly, two of the included studies were showed a statistically significant association between diabetic foot ulcer and callus of the feet. Diabetic patients who had a callus on their foot was 12.67 times more likely to develop foot ulcer as compared to diabetic patients who had no callus on their feet (OR = 12.67, 95%, CI: 6.47–24.79) (Fig. 5).

#### Association between diabetic foot ulcer and body mass index (BMI)

**Fig. 6:**
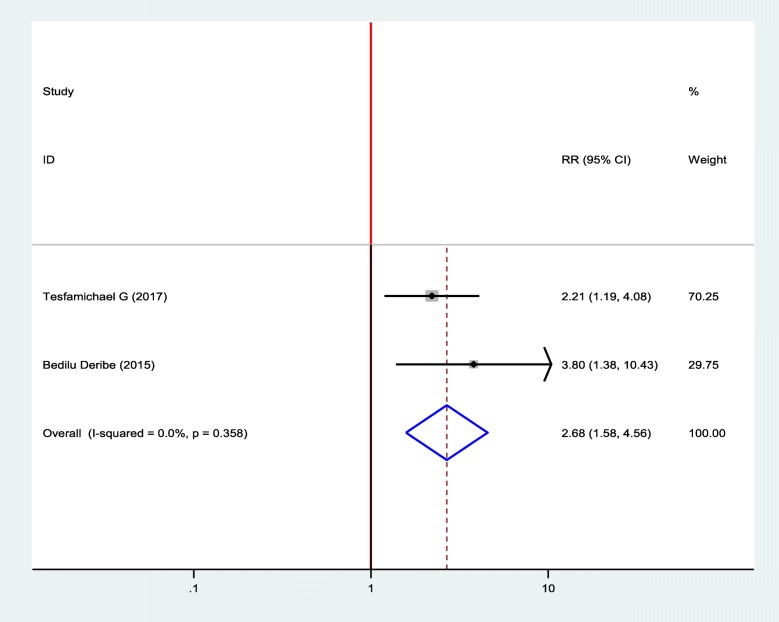
Forest plot of association between diabetic foot ulcer and BMI in Ethiopia, 2019

Two studies were selected to show the association between diabetic foot ulcers and BMI, and two of them showed a positive association between BMI and diabetic foot ulcers [18, 19]. Diabetic patients who had BMI greater than or equal 24.5 kg/m^2^ were 2.68 times more likely to develop foot ulcer as compared to diabetic patients who had BMI less than 24.5 kg/m^2^ (OR = 2.68, 95%, CI: 1.58–4.56) (Fig. 6).

#### Association between diabetic foot ulcer and DM self- care practice

**Fig. 7:**
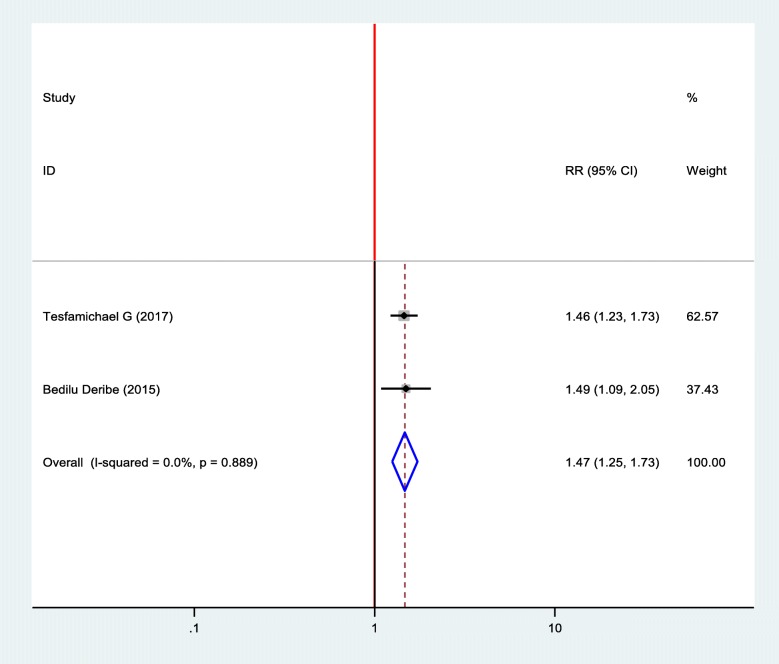
Forest plot of association between diabetic foot ulcer and health care practice in Ethiopia, 2019

Two studies were included in the meta-analysis to show the association between diabetic foot ulcer and self-care practice of the patients and two of the included studies showed statistical significance between diabetic foot ulcer and self- care practice [18, 19]. The finding revealed that the odds of developing foot ulcer was 1.47 times more likely among patients who had poor self- care practice than patients who had good health care practice (OR = 1.47, 95%, CI: 1.25–1.73) (Fig. 7).

#### Association between diabetic foot ulcer and sex

**Fig. 8:**
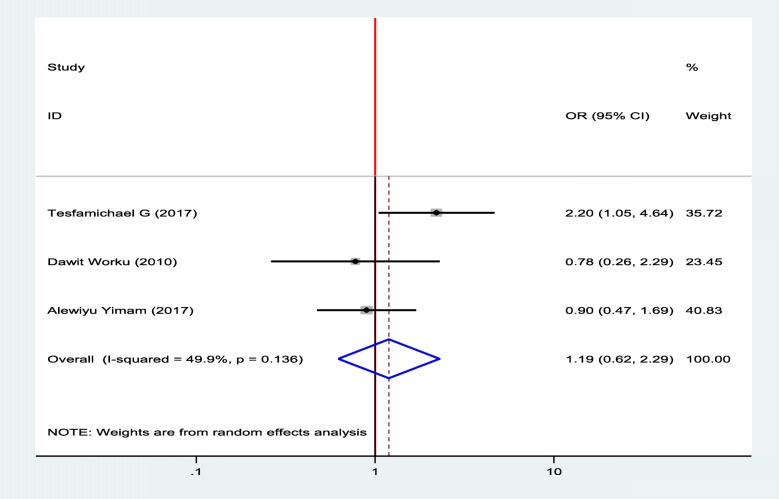
Forest plot of association between diabetic foot ulcer and sex in Ethiopia, 2019

To show the association between diabetic foot ulcer and sex of patients, three studies were selected for meta-analysis [12, 15, 19]. One study showed, there was statistically significant association between diabetic foot ulcer and sex and two studies showed that there was no significant association between diabetic foot ulcer and sex of the patients [12, 15], however, there was no significant association between diabetic foot ulcer and sex of the patients from their pooled findings (Fig. 8).

#### Association between diabetic foot ulcer and types of DM

**Fig. 9:**
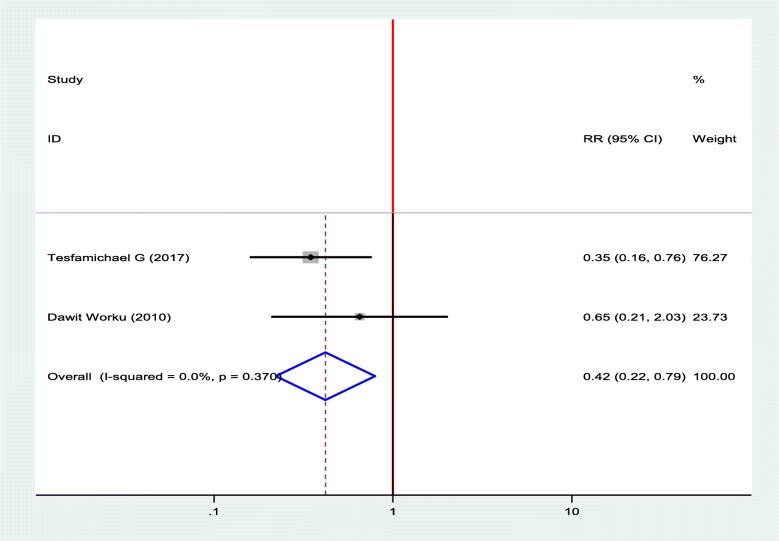
Forest plot of association between diabetic foot ulcer and types of DM in Ethiopia, 2019

To compute the association between diabetic foot ulcers and types of DM, two studies were selected for meta-analysis [12, 19]. The pooled result of the analysis showed that there was a statistically significant association between diabetic foot ulcers and types of DM. Type I DM decrease the odds of developing diabetic foot ulcer by 58% as compared to type II DM (OR = 0.42, 95%, CI: 0.22–0.79) (Fig. 9).

#### Association between diabetic foot ulcer and duration of DM

**Fig. 10:**
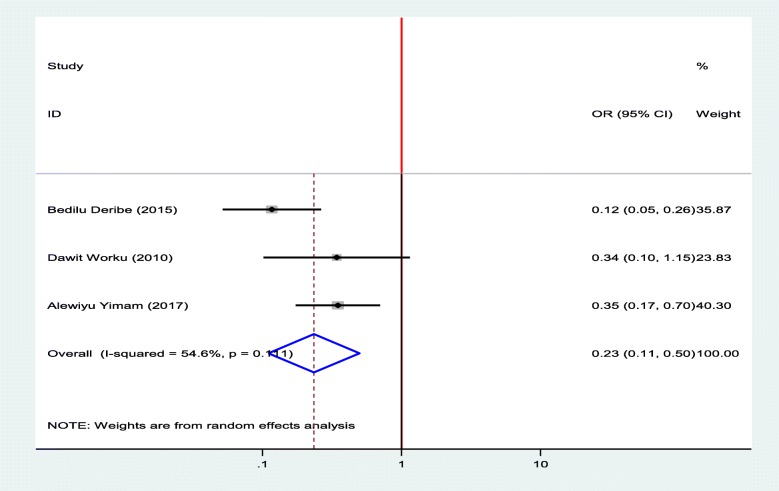
Forest plot of association between diabetic foot ulcer and duration of DM in Ethiopia, 2019

To identify the association between diabetic foot ulcer and the duration of patients stayed with DM, three studies were selected for meta-analysis [12, 15, 18]. The pooled finding showed that duration of the patients stayed with DM were significantly associated with diabetic foot ulcer. Being diabetic patients for less than 10 years decrease the odds of developing diabetic foot ulcer by 77% as compared to diabetic patients who stayed with DM for ≥ 10 years (OR = 0.23, 95%, CI: 0.11–0.50) (Fig. 10).

#### Association between diabetic foot ulcer and the age of the patients

**Fig. 11:**
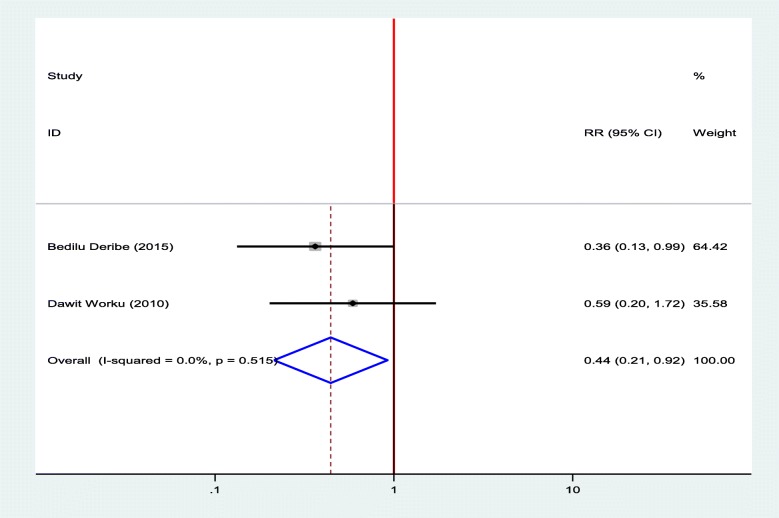
Forest plot of association between diabetic foot ulcer and age of the patients in Ethiopia, 2019

Two studies were selected for meta-analysis to observe the association between diabetic foot ulcer and the age of the patients [12, 18]. The pooled finding showed that patients who were aged less than 45 were decreased the odds of developing diabetic foot ulcers by 56% as compared to patients who were aged > = 45 years (OR = 0.44, 95%, CI: 0.21–0.92) (Fig. 11).

## Discussion

To the best of our knowledge, this meta-analysis and systematic review are the first of its kind that conducted at the national level to estimates prevalence and identifies factors associated with a diabetic foot ulcers in Ethiopia. Even though the prevalence of diabetic foot ulcers differs from region to region, this study depicted that the pooled prevalence of diabetic foot ulcers in Ethiopia is 12.98%. The difference in prevalence from region to region may be due to the differences in sample size and year of study. In Ethiopia, the prevalence of diabetic foot ulcers is relatively increasing from previous studies to recent ones. This relative increment from previous studies to recent might be due to the change in the lifestyle of people toward a sedentary way of life. The pooled prevalence of diabetic foot ulcers in Africa is 13.0% that is almost the same as Ethiopia [28]. The prevalence of diabetic foot ulcers in this meta-analysis is lower than a study conducted in Khartoum Sudan, 18.1% [29] and Spain 17.4% [30]. This discrepancy could be due to the difference in the study method.

This systematic review and meta-analysis also identified factors associated with a diabetic foot ulcers. Being a rural residence is one of the factors that had a positive association with a diabetic foot ulcer. This finding is supported by a study conducted in a developing country [31]. The possible reason for those diabetic patients who reside in rural areas had poor awareness about self-care practice and most of the diabetic patients from a rural area in Ethiopia are farmers and walk by their barefoot, hence they are subjected to bite and injury. Bites and injury to the feet might result in ulceration of the feet and poor healing process of the wound. Moreover, inadequate access to information that can help them to give self-care to reduce diabetic foot ulcer and they are less likely to take care of their foot problems. This, in turn, exposes the patient’s feet to develop foot ulcers.

The self- care practice of people with diabetes mellitus is different from person to person. The finding from this systematic review and meta-analysis showed that a good self-care practice is protective for the occurrence of diabetic foot ulcers. This finding is comparable with the previous study conducted in different settings [32–34]. Poor practicing foot self-care increase the occurrence of diabetic foot ulcer due to the absence of washing their feet daily, lack of drying appropriately after washing, and lack early management of any abnormality that may occur on the foot.

The result of this study also indicates that the duration that a patient stayed with the diabetic disease is one of the risk factors for the development of foot ulcers. As the time patient lives with the diabetic Mellitus increase the chance of occurrence of diabetic foot ulcer will also increase. This is due to the disease condition to increase its severity from time to time if not adequately controlled. This finding is in line with other studies that were, as the duration of patients live with diabetic Mellitus increase, the occurrence of diabetic foot ulcer also increased [35–46].

The presence of callus on the feet was another risk factor for the occurrence of diabetic foot ulcer and it is consistent with previous studies conducted in different settings [31, 43, 47]. This might be due to the decreament of the blood supply to the area and if an injury occurred in this area, the chance of healing is rare. In diabetic patients, the callus on the feet develops due to peripheral neuropathy. Neuropathy leads to deformity and lack of sensation, which results in persistent abnormal pressure on the foot. The cells of skin react to it by increasing keratinization and turn into a callus, which predisposes patients to develops diabetic foot ulcers.

The other finding from this study is that increased body mass index is positively associated with a diabetic foot ulcers. Most of the time, an increase in body mass index is associated with the incensement of obesity. As obesity increase, atherosclerosis will be increased and this, in turn, decreases blood supply to lower extremities so that the environment will be good for the growth of bacteria if a wound occurs. Many previous research findings also revealed that increment in body mass index is a risk for diabetic foot ulcers [36, 39, 44, 48].

### Limitation of the study

We tried to use comprehensive search strategies for this systematic review and meta-analysis by including both published and unpublished studies. A random-effects model was used to address the potential variability across studies. However, the restriction of studies written in English limited the number of studies included in a meta-analysis. In the current meta-analysis, almost all included studies were conducted at the large towns of the country, which could not be representative of all districts of the country. In addition, the small sample due to a limited number of included studies with a small sample size for all included studies was another limitation of this study. In addition, all of the studies included in this review were cross-sectional study design; as a result, the outcome variable might be affected by other confounding variables, decreases the power of the study and it decreases causal conclusion between foot ulcer and factors associated with a foot ulcer.

## Conclusion

In current systematic review and meta-analysis, the prevalence of diabetic foot ulcers in Ethiopia is relatively low in comparison with developed countries. This study showed that being older age, duration of living with the disease for a longer time, being a rural residence, high BMI, presence callus on the feet, poor self- care practice of patients were positively associated with the development of diabetic foot ulcers. Therefore, appropriate interventions towards patient self-care practice, lifestyle modification, and continuous follow up are vital to prevent diabetic foot ulcers. Health care professionals have to play their role in tackling diabetic foot ulcers through proper health education and treatment of patients.

## Data Availability

All data generated or analysed during this study are included in this published article.

## Abbreviations

BMI: Body Mass Index
CI: Confidence Interval
DFU: Diabetic Foot Ulcer
DM: Diabetes Mellitus
OR: Odd Ratio
SNNP: Southern Nation Nationalities and People
